# Supplementary material for: Integrating Clinical and Imaging Markers for Survival Prediction in Advanced NSCLC Treated with EGFR-TKIs
Source: Cancers (Basel). 2025 Aug 3;17(15):2565. doi: 10.3390/cancers17152565 (PMC12345999; doi:10.3390/cancers17152565)
Supplement: Supplementary file 1 [file cancers-17-02565-s001.zip › cancers-3748216-supplementary.pdf]

Supplementary Table Legends

Supplementary Table S1. Sample size calculation per group.

Supplementary Table S2. Mutational subtypes.

Supplementary Table S3. Estimated log hazard ratios in the full and reduced multivariable flexible parametric regression models.

Supplementary Table S4: Estimated hazard ratios in the full and reduced multivariable flexible parametric regression models using complete case analysis.

Supplementary Table S5. Performance measures from internal validation of 200 bootstrap samples.

Supplementary Table S6. Performance measures from internal validation of 200 bootstrap samples using complete case analysis.

Supplementary Table S7. Demonstration of the model-estimated survival probability at each time point from nine sample patient, estimated from complete case analysis.

Supplementary Table S1. Sample size calculation per group

Variables	HR	Ratio (N2/N1)	N1	N2	Total
Albumin-to-globin ratio [32]	1.88	1	43	43	86
COPD [14]	1.52		93	93	186
Neutrophil-to-Lymphocyte Ratio (NLR ≥ 5) [31]	3.002		16	16	32
Sarcopenia [16]	2.23		28	28	56
Blood urea nitrogen/albumin ratio (>0.80) [43]	0.59		60	60	120

Supplementary Table 2. Mutational subtypes

Mutation subtypes	Total, N (%)	Missing, N (%)	Hazard ratio (95%CI)	6-month survival, % (95%CI)	12-month survival, % (95%CI)	18-month survival, % (95%CI)	p-value
Mutation investigated	188 (99.47)	1 (0.53)					
Exon 19 deletion	120 (63.49)	0	0.97 (0.62-1.50)	86.67 (79.16-91.61)	73.33 (64.46-80.33)	55.83 (46.50-64.18)	0.874
Exon 21	0	0	NA	NA	NA	NA	NA
L858R	54 (28.57)	0	0.98 (0.61-1.58)	87.04 (74.72-93.60)	77.78 (64.20-86.72)	55.56 (41.39-67.59)	0.940
L861Q	9 (4.76)	0	1.18 (0.43-3.22)	66.76 (28.17-87.83)	55.56 (20.42-80.45)	55.56 (20.42-80.45)	0.744
Exon 20	3 (1.59)	0	0.59 (0.08-4.26)	100 (NA)	100 (NA)	66.67 (5.41-94.52)	0.593
T790M	5 (2.65)	0	0.97 (0.24-3.95)	80.00 (20.38-96.92)	60.00 (12.57-88.18)	60.00 (12.57-88.18)	0.968
S768I	1 (0.53)	0	3.35 (0.46-24.26)	100 (NA)	NA	NA	0.199
Exon 18	3 (1.59)	0	0.72 (0.10-5.18)	100 (NA)	66.67 (5.41-94.52)	66.67 (5.41-94.52)	0.740

Supplementary Table S3. Estimated log hazard ratios in the full and reduced multivariable flexible parametric regression models.

Predictors	Full model			Reduced model		
	Beta	95%CI	P value	Beta	95%CI	P value
**BMI group**						
< 18.5	0.302	-0.293, 0.898	0.320	0.294	-0.277, 0.867	0.313
18.5 – 22.9	0.000	Reference	NA	0.000	Reference	NA
≥ 23	-0.552	-1.091, -0.012	**0.045**	-0.649	-1.170, -0.128	**0.015**
**Contralateral lung metastasis**						
No	0.000	Reference	NA		Not included	
Yes	-0.461	-0.961, 0.040	0.071			
**Bone metastasis**						
No	0.000	Reference	NA	0.000	Reference	NA
Yes	0.656	0.196, 1.116	**0.005**	0.734	0.289, 1.179	**0.001**
**Hemoglobin (g/dl)**	-0.074	-0.218, 0.069	0.310		Not included	
**PMN (%)**	0.010	-0.021, 0.041	0.535		Not included	
**PLR group**						
PLR ≤ 200	0.000	Reference	NA		Not included	
PLR > 200	0.031	-0.489, 0.550	0.908			
**NLR group**						
NLR < 5	0.000	Reference	NA	0.000	Reference	NA
NLR ≥ 5	0.508	-0.181, 1.198	0.148	0.812	0.331, 1.292	**0.001**
**Cr**	-0.536	-1.411, 0.340	0.230		Not include	
**AGR group**						
AGR ≥ 1	0.000	Reference	NA	0.000	Reference	NA
AGR < 1	0.671	0.190, 1.151	**0.006**	0.773	0.336, 1.210	**0.001**
**MPA group**						
MPA < 29 (mm)	0.000	Reference	NA	0.000	Reference	NA

Predictors	Full model			Reduced model		
	Beta	95%CI	P value	Beta	95%CI	P value
MPA ≥ 29 (mm)	0.945	0.470, 1.420	**<0.001**	1.008	0.544, 1.471	**<0.001**
**Restricted Cubic Splines**						
RCS1	2.134	0.974, 3.294	**<0.001**	2.126	0.968, 3.284	**<0.001**
RCS2	1.122	0.119, 2.126	**0.028**	1.106	0.104, 2.108	**0.030**
RCS3	-3.327	-6.090, -0.564	**0.018**	-3.277	-6.035, -0.519	**0.020**
**Model Intercept (constant)**	-5.647	-8.767, -2.526	**<0.001**	-6.505	-8.104, -4.906	**<0.001**

Abbreviations: CI, confidence interval; NA, not available; RCS, restricted cubic spline function

Supplementary Table S4. Estimated hazard ratios in the full and reduced multivariable flexible parametric regression models using complete case analysis.

Predictors	Full model CCA (n=172)			Reduced CCA (n=173)		
	HR	95%CI	P value	HR	95%CI	P value
BMI group						
< 18.5	1.38	0.75-2.55	0.297	1.40	0.77-2.52	0.266
18.5 – 22.9	1.00	Reference	NA	1.00	Reference	NA
≥ 23	0.51	0.28-0.84	0.029	0.47	0.26-0.84	0.011
Contralateral lung metastasis						
No	1.00	Reference	NA		Not include	
Yes	0.66	0.39-1.11	0.116			
Bone metastasis						
No	1.00	Reference	NA	1.00	Reference	NA
Yes	1.84	1.10-3.06	0.020	2.00	1.22-3.28	0.006
Hemoglobin (g/dl)	0.91	0.79-1.06	0.250		Not include	
PMN (%)	1.01	0.98-1.05	0.417		Not include	
PLR group						
PLR ≤ 200	1.00	Reference	NA		Not include	
PLR > 200	1.19	0.69-2.08	0.529			
NLR group						
NLR < 5	1.00	Reference	NA	1.00	Reference	NA
NLR ≥ 5	1.61	0.77-3.38	0.208	2.43	1.44-4.08	0.001
Cr	0.65	0.27-1.55	0.332		Not include	
AGR group						
AGR ≥ 1	1.00	Reference	NA	1.00	Reference	NA
AGR < 1	2.01	1.21-3.34	0.007	2.35	1.47-3.77	<0.001
MPA group						
MPA < 29 (mm)	1.00	Reference	NA	1.00	Reference	NA
MPA ≥ 29 (mm)	2.84	1.70-4.74	<0.001	3.14	1.91-5.16	<0.001

Supplementary Table S5. Performance measures from internal validation of 200 bootstrap samples.

Performance measures	Original apparent performance	Test performance	Optimism
C-statistics	0.75<br>(95%CI 0.74 - 0.75;<br>Min 0.68; Max 0.83)	0.71<br>(95%CI, 0.71 - 0.71;<br>Min 0.60; Max 0.74)	0.038<br>(95%CI, 0.03-0.04;<br>Min -0.032; Max 0.23)
Somers' D	0.50<br>(95%CI, 0.49-0.51;<br>Min 0.36; Max0.67)	0.42<br>(95%CI, 0.41-0.43;<br>Min 0.20; Max 0.47)	0.08<br>(95%CI, 0.07-0.09;<br>Min -0.06; Max0.46)
Royston R^2_D	0.42<br>(95%CI, 0.41-0.43;<br>Min 0.22; Max 0.64)	0.30<br>(95%CI, 0.30-0.31;<br>Min 0.10; Max 0.38)	0.12<br>(95%CI 0.11-0.13;<br>Min -0.09; Max 0.54)
Royston D	1.77<br>(95%CI; 1.73-1.81;<br>Min 1.09; Max 2.70)	1.35<br>(95%CI, 1.33-1.38;<br>Min 0.68; Max 1.61)	0.41<br>(95%CI 0.37-0.46;<br>Min -0.28; Max 2.02)

Supplementary Table S6. Performance measures from internal validation of 200 bootstrap samples using complete case analysis.

Performance measures	Original apparent performance	Test performance	Optimism
C-statistic	0.76<br>(95%CI 0.76 - 0.77;<br>Min 0.67; Max 0.86)	0.72<br>(95%CI, 0.71 - 0.72;<br>Min 0.60; Max 0.75)	0.047<br>(95%CI, 0.041-0.053;<br>Min -0.049; Max 0.241)
Somers' D	0.525<br>(95%CI, 0.517-0.534;<br>Min 0.346; Max0.729)	0.431<br>(95%CI, 0.423-0.439;<br>Min 0.204; Max 0.499)	0.094<br>(95%CI, 0.082-0.106;<br>Min -0.097; Max0.439)
Royston R^2_D	0.467<br>(95%CI, 0.456-0.478;<br>Min 0.195; Max 0.744)	0.322<br>(95%CI, 0.311-0.332;<br>Min 0.103; Max 0.433)	0.145<br>(95%CI 0.130-0.161;<br>Min -0.071; Max 0.630)
Royston D	1.940<br>(95%CI; 1.894-1.986;<br>Min 1.006; Max 3.490)	1.413<br>(95%CI, 1.378-1.448;<br>Min 0.693; Max 1.790)	0.527<br>(95%CI 0.468-0.586;<br>Min -0.224; Max 2.757)

Supplementary Table S7. Demonstration of the model-estimated survival probability at each time point from nine sample patient, estimated from complete case analysis.

Input predictors						Model estimation of survival probability (%, 95%CI)		
No	BMI groups	Bone metastasis	NLR>5	AGR<1	MPA≥29	6 months	12 months	18 months
1	≥ 23	No	No	No	No	98.44<br>(96.54-99.30)	95.53<br>(91.22-97.75)	89.92<br>(0.02-94.40)
2	18.5-22.9	No	No	No	No	96.74<br>(93.61-98.35)	90.78<br>(84.12-94.73)	79.86<br>(68.70-87.40)
3	<18.5	No	No	No	No	95.47<br>(89.72-98.04)	87.35<br>(75.21-93.78)	73.03<br>(53.37-85.44)
4	18.5-22.9	No	No	No	Yes	90.10	73.79	49.34

Input predictors						Model estimation of survival probability (%, 95%CI)		
No	BMI groups	Bone metastasis	NLR>5	AGR<1	MPA≥29	6 months	12 months	18 months
						(81.76-94.75)	(58.23-84.29)	(29.15-66.70)
5	18.5-22.9	Yes	Yes	No	No	85.14 (71.97-92.43)	62.54 (40.21-78.53)	33.60 (12.42-56.54)
6	<18.5	No	Yes	Yes	No	76.74 (56.10-88.58)	46.20 (20.89-68.34)	16.62 (2.78-40.71)
7	<18.5	No	No	Yes	Yes	70.97 (48.64-84.94)	36.78 (14.17-59.59)	9.78 (1.08-30.31)
8	18.5-22.9	Yes	Yes	Yes	No	68.48 (46.51-82.92)	33.14 (11.74-56.58)	7.68 (0.65-26.99)
9	≥ 23	Yes	Yes	Yes	Yes	56.98 (27.84-78.08)	19.38 (2.71-47.41)	2.21 (0.02-18.23)
